# A Paper on Diseases of the Skin

**Published:** 1841-01-02

**Authors:** C. N. Berkeley


### MEDICAL EXAMINER.

DEVOTED TO MEDICINE, SURGERY, AND THE COLLATERAL SCIENCES.

No. 1.] PHILADELPHIA, SATURDAY, JANUARY 2, 1841. [Vol. IV.

### ORIGINAL COMMUNICATIONS.

*A Paper on Diseases of the Skin.* By C. N. Berkeley, M. D.

The present state of our knowledge of diseases of the skin presents a striking contrast with that of other affections, both medical and surgical. While pathological investigations have been pursued with interest, and daily additions thus made to accurate diagnosis and treatment of disease in almost every form, affections of the skin, the most palpable lesions of the system, and those most evidently challenging the notice of the practitioner, have in this country been so much neglected, as in part to be thrown out of regular practice, and consigned to the care of empyrics. Reasons for this are offered, such as the comparative infrequency of occurrence of cutaneous diseases, the habit of domestic treatment in their mild forms, and their intractable nature, when brought to the physician in the chronic or inveterate stages, etc. But, perhaps, a better excuse may be found in the difficulties supposed to attend their study, from the want of systems of classification and nomenclature adapted to these diseases, as presented in the United States. There is certainly much weight in this; for, besides the objection on the ground of the expense of importing the French and English works, the diseases they describe differ so much in variety and extent from those occurring with us, as to offer strong difficulties in the way of a just comparison, thus rendering these works of less practical value than their intrinsic merits would indicate.

These difficulties may be obviated, we think, at least for practical purposes, by assuming some plan, based upon the classifications of foreign writers, which shall embrace most of the diseases they describe, with those of our own country, but so arranged as to correct in part the confusion of a multiplicity of synonymes, and at the same time indicate the general nature of each class and variety of disease. Such a plan is now offered—not to the scientific inquirer, who will, of course, refer to the authors themselves from whose works it is partly drawn—but to the student whose opportunities do not permit access to them, and to the practitioner whose engagements will not allow him the time necessary for their study and comprehension.

To establish a classification on a strictly *scientific basis*, we should assume for it the anatomical lesions and pathological phenomena of the diseases to be arranged. This has been attempted in those of the skin, but with little success. To comprehend the difficulty of such a plan, we must investigate the special anatomy of the skin: we give it very briefly, as demonstrated by Messrs. Breschet and Roussel de Vauzeme. According to their observations the skin consists of—1st, the *dermis*, enveloping the capillaries, the lymphatics, the nervous filaments, and the parenchyma of other organs contained in its substance.

2d. The *papillæ*, or organs of touch, the extremities of the nervous filaments.

3d. The perspiratory apparatus, or organ of secretion and excretion of perspiration.

4th. The apparatus of inhalation, or absorbent canals.

5th. The apparatus producing the mucous matter.

6th. The apparatus producing the colouring matter.

Some doubts exist as to the accuracy of these investigations, since Chevalier under similar circumstances arrived at very different results; still we will assume them as nearly correct, some such organization being necessary to account for the various functions of the skin. Our object in bringing them forward is to show the almost impossibility of establishing a pathological arrangement of cutaneous diseases,—that is, of assigning to each of them a cause, and a location in an abnormal condition of one or the other of these tissues. How could inflammation exist in the perspiratory apparatus without involving that of inhalation and absorption; or how could the minute vessels of the mucous tissue be affected without implicating those of the colouring matter? for the space occupied by each of these systems is so small as to defy detection with the naked eye, requiring the assistance of the most powerful glasses to render them evident to the senses. Daily experience shows the impossibility of inflammation existing in one or the other organ of the skin for a considerable time, without extending to those adjacent. Thus, in eczema, the most common vesicular affection of the skin, the first and simplest form presents a slight elevation of the cuticle, filled with clear serum, and apparently without inflammation; a degree farther we find a partial change in the character of the serum, and evidences of inflammation as in eczema rubrum. Still farther, we have the serum entirely altered, becoming purulent, and covering the surface, with scabs as in eczema impetiginodes, which soon becoming pustular presents a genuine impetigo. The same condition prevails in scabies, which, if neglected, at different periods of its duration, exhibits every variety of cutaneous disease, from the simple vesicle, its type and origin, to the severest forms of pustular and papular affection, followed by universal desquamation. These facts illustrate the position assumed, and warrant, we think, an attempt at some simpler and more practical classification.

In the following plan a distinction is drawn between syphilitic and venereal cutaneous affections; this is not the proper place to give the reasons for such a distinction. Syphilis is used to express *chancre* and its *special* consequences; *venereal* implies all other diseases, the results of *impure coition*, not dependent on chancre. We propose to follow Plumbe in part, arranging cutaneous diseases under two general divisions. First, those of local origin, or dependent on the skin alone: second, those of a constitutional character, produced by some cause affecting the general system and developed through the skin.

First Division.—Diseases of the skin proper, or those originating in the skin, independent of, though modified by, constitutional causes.

| | *Synonymes.* | | |
|---|---|---|---|
| Pustular Diseases | Varus, Gutta rosea, Dartre pustuleuse, Couperose, Copper nose, Couperose | Acne | A. mentior sycosis—probably contagious. A. punctata, A. syphilitica, A. indurata, A. sebacea (Generally chronic), A. rosacea, acute — Non-contagious. |
| | Tinea-Teigne, Favus, P. lupinosa | Porrigo | P. favosa — Contagious when originating as porrigo around the bulbs of the hair; when |
| | Ringworm | | P. scutulata — when consequent on impetigo, probably non-contagious, though followed by permanent baldness in the affected part. |
| | Agria, Scabies fera, Furunculi Atonici | Ecthyma | Non-contagious; sometimes complicated with syphilis; chronic. |
| Vesicular | Dartre squammeuse, Humid tetter, Running scall | Eczema | E. simplex, or solare, closely analogous to lichen tropicus, or prickly heat; E. rubrum—often periodical; usually the origin of chronic E. in debilitated constitutions; E. impetiginodes—apt to lose its vesicular form, and become impetigo — Non-contagious. |
| | Itch, Psora-gale, Rogne | Scabies | Generally acute and contagious; when chronic, it loses its vesicular character, and becomes either pustular, squamous, or papular; in the latter form closely resembling prurigo senilis. Scabies is produced by the acanus scabei. |
| Squamous | Scabies sicca, Dartre furfuracée arrondie, Dartre ecailleuse, Dartre squammeuse lichenoide, Dry Scall, Scaly Leprosy, Scaly Tetter | Psoriasis | P. guttata, P. labialis (Generally acute); P. circinnata, or lepra, P. diffusa, P. inveterata, P. preputialis, P. palmaris, grocers' or bakers' itch (Chronic) — Non-contagious. |
| | Dandriff, Dartre furfuracée—volante, Lichen | Pityriasis | P. capitis—chronic; P. versicolor, P. rubra (usually acute) — Non-contagious and rarely the subject of medical treatment. |
| | Fish Skin disease | Icthyosis | Chronic and non-contagious. |

| | | | | |
|---|---|---|---|---|
| BULLOUS | *Synonymes.*<br>Ulcus Atonicum | Rupia | R. simplex; acute and non-contagious. | |
| TUBERCULAR | Herpes exedens<br>Lupus vorax<br>Dartre rongeante | Lupus | L. exedens<br>L. syphiliticus<br>L. non exedens | Non-contagious. |
| | Cancroide<br>Keloide | Cheloidea | Chronic and non-contagious. | |
| MACULÆ | Mother mark<br>Spilus<br>Mole | Naevi | Naevi vasculares. | |

SECOND DIVISION.—Diseases produced by causes acting through the constitution.

| | | | | |
|---|---|---|---|---|
| PUSTULAR | *Synonymes.*<br>Crusted tetter<br>Dartre crustacée<br>Running tetter<br>Melitagre | Impetigo figurata<br>Imp. sparsa | Porrigo larvalis<br>Tinea mucosa<br>Crusta lactea<br>Teigne<br>Dartre crustacée flavescente | Non-contagious, and frequently salutary in its influence, especially in children, during the summer and winter. |
| | | Venereal & syphilitic | The most common form of venereal and syphilitic cutaneous affection. | |
| | Glanders<br>Farcy glanders | Equinia | E. glandulosa—acute glanders<br>Farcy glanders, generally chronic | Contagious. |
| VESICULAR | | Eczema | E. of the scalp, confounded with porrigo<br>Chronic E.<br>E. of the face, mistaken frequently for crusta lactea, or impetigo | Non-contagious, and perhaps salutary. |
| | | Syphilitic & venereal | Very rare—chronic. | |
| | *Synonymes.*<br>Tetter<br>Dartre<br>Olophlyctide | Herpes | H. phlyctenodes<br>H. labialis<br>H. preputialis<br>H. zoster<br>H. iris (Generally acute.)<br>H. circinnatus—evanestent ringworm, liable to become chronic | Non-contagious. |
| SQUAMOUS | | Syphilitic eruptions | Unconnected with other forms of cutaneous disease, and perhaps entirely dependent on constitutional syphilis. | |
| PAPULAR ERUPTIONS. | *Synonymes.*<br>Papula<br>Scabris sicca<br>Gall Leche<br>Dartre furfuracée volante | Lichen | L. simplex<br>L. strophulus—red gum<br>L. urticatus<br>L. agrius<br>L. syphiliticus—generally of venereal origin | Acute—non-contagious. |
| | Pruritus<br>Cresmos<br>Scabies papuliformis | Prurigo | P. mitis—acute.<br>P. formicans<br>P. senilis<br>P. podicis<br>P. genitalium | Generally chronic and non-contagious; in the form of P. senilis, perhaps salutary, especially in females, after their "change in life." |
| | | Syphilitic | Generally result of constitutional syphilis. | |

| Class | *Synonymes.* | Disease | Varieties | Character |
|---|---|---|---|---|
| EXANTHEMES | Epera aspretudo<br>Nettle rash<br>Purpura urticata<br>Febris urticata<br>Exanth-urticatum | Urticaria | U. febritis—acute<br>U. evanida } Chronic generally.<br>U. tuberosa } | Non-contagious. |
| | Intertrigo<br>Tooth rash<br>Gum | Erythema | E. fugax<br>E. papulatum<br>E. Nodosum | Acute—non-contagious. |
| | | Erysipelas | Acute<br>Chronic | Non-contagious; sometimes epidemic in hospitals. |
| | Rose rash<br>Rosacea<br>Rubeola<br>Spuria | Roseola | R. infantilis<br>R. annulata<br>R. aestiva<br>R. autumnalis | Acute and non-contagious. |
| | | | R. syphilitica | A primary affection, usually coincident with gonorrhœa; at times chronic. |
| MACULÆ | Land scurvy<br>Petechia<br>Peliose | Purpura | P. simplex<br>P. hæmorrhagia | Acute—non-contagious. |
| | | | P. syphilitica | Acute and chronic; perhaps purely of syphilitic origin. |
| | Liver spots<br>Tâches hepatiques<br>Cloasma | Ephelis | | Generally chronic; non-contagious; apt to accompany pregnancy, and retire with its cessation. |
| BULLOUS | Febris bullosa<br>Pompholix<br>Dartre phlyctenoide confluente | Pemphigus | P. acute } Acute<br>Pomphol-solitarius }<br>Pomph-dintinus—chronic pemphigus | Non-contagious. |
| | Ulcus atonicum | Rupia | R. prominens<br>R. escharotica<br>R. syphilitica | Chronic; non-contagious. |
| TUBERCULAR | | Elephantiasis | | Chronic—non-contagious. |
| | | Syphilitic tubercle | | Generally of syphilitic origin—chronic. |
| UNCLASSIFIED DISEASES. | Framboesia<br>Mollusum<br>Lepoides<br>Melanosis | | | |
| | Furuncle | | | Not properly a disease of the skin, but of the sub-cutaneous cellular tissue. |
| | Scrofula | | | Incapable perhaps of originating, yet complicating and modifying all the diseases embraced in the second division. |

Variola, varioloid, and vaccina; varicella, with military fever; rubeola and scarlatina, are omitted. With the exception of vaccina, they are essentially fevers, and belong to works on general practice. Vaccina, if classified, would be considered as confined to the skin proper; it is not introduced on account of its intimate relations with variola.

